# Intergenerational transmission: Theoretical and methodological issues and an introduction to four Dutch cohorts

**DOI:** 10.1016/j.dcn.2020.100835

**Published:** 2020-08-08

**Authors:** Susan Branje, Sanne Geeraerts, Eveline L. de Zeeuw, Anoek M. Oerlemans, M. Elisabeth Koopman-Verhoeff, Susanne Schulz, Stefanie Nelemans, Wim Meeus, Catharina A. Hartman, Manon H.J. Hillegers, Albertine J. Oldehinkel, Dorret I. Boomsma

**Affiliations:** a Youth and Family, Department of Educational and Pedagogical Sciences, Utrecht University, Utrecht, the Netherlands; b Netherlands Twin Register, Department of Biological Psychology, Vrije Universiteit Amsterdam, Amsterdam, the Netherlands; c Department of Psychiatry, University of Groningen, University Medical Centre Groningen, Groningen, the Netherlands; d Department of Child and Adolescent Psychiatry/Psychology, Erasmus MC University Medical Center Rotterdam-Sophia Children’s Hospital, Rotterdam, the Netherlands; e The Generation R Study Group, Erasmus MC University Medical Center Rotterdam, Rotterdam, the Netherlands

**Keywords:** Intergenerational transmission, Gene-environment, Parent, Children, Longitudinal

## Abstract

Behaviors, traits and characteristics are transmitted from parents to offspring because of complex genetic and non-genetic processes. We review genetic and non-genetic mechanisms of intergenerational transmission of psychopathology and parenting and focus on recent methodological advances in disentangling genetic and non-genetic factors. In light of this review, we propose that future studies on intergenerational transmission should aim to disentangle genetic and non-genetic transmission, take a long-term longitudinal perspective, and focus on paternal and maternal intergenerational transmission. We present four large longitudinal cohort studies within the Consortium on Individual Development, which together address many of these methodological challenges. These four cohort studies aim to examine the extent to which genetic and non-genetic transmission from the parental generation shapes parenting behavior and psychopathology in the next generation, as well as the extent to which self-regulation and social competence mediate this transmission. Conjointly, these four cohorts provide a comprehensive approach to the study of intergenerational transmission.

## 1 Introduction

Psychopathology and parenting behavior are transmitted across generations. Intergenerational transmission, or the extent to which behaviors and characteristics of individuals from one generation are recurring in offspring, has been reported for multiple characteristics, such as personality and psychopathology, educational attainment and Socioeconomic Status (SES), Body Mass Index (BMI), life style factors, and health, as well as relational processes, such as quality of parenting or divorce (Thornberry et al., 2003; Eaves et al., 1999). This transmission from parents to offspring occurs because of complex processes that involve genetic and non-genetic factors. This last class of factors is referred to as environmental, and in the context of non-genetic transmission from one generation to the next also as ‘cultural transmission’ (Cavalli-Sforza and Feldman, 1973; Eaves, 1976).

This review aims to consider the mechanisms for intergenerational transmission of psychopathology and parenting behavior. We also note that the intergenerational transmission of parenting behavior and psychopathology involves reciprocal processes in which parenting can be an important mechanism in the transmission of psychopathology and vice versa. We will discuss some methodological considerations that need to be taken into account when studying transmission from one generation to the next, with a focus on transmission from parents to offspring. In addition, this paper introduces four large Dutch longitudinal cohort studies that aim to examine intergenerational transmission, and explains how these cohort studies take the methodological considerations for studying intergenerational transmission into account. Within the Consortium on Individual Development, we aim to examine the extent to which genetic and non-genetic transmission between generations create differences between children and adolescents in parenting and psychopathology, as well as the extent to which, self-regulation and social competence mediate this transmission. To this end, four large cohort studies in the Netherlands that consists of 2- or 3 generation families have joined forces to collect longitudinal data across multiple domains of life, as well as biomarker and biological information from DNA variants and epigenetic information.

## 2 Intergenerational transmission

Intergenerational transmission describes the intergenerational continuity within, and possibly also across behaviors and characteristics. Broadly speaking, intergenerational transmission can arise when a parental trait affects the trait in their children via genetic inheritance, through the transmission of DNA, via cultural transmission, or through the interplay between genetics and the environment. Intergenerational transmission can be reflected in homotypic continuity, affecting the same behavior or trait across generations, or heterotypic continuity, affecting different traits across generations. According to Cavalli-Sforza and Feldman (1973), cultural transmission occurs when parents (and other group members) influence a child's behavior. In this paper we follow their example and discuss genetic and cultural transmission from parent to child, omitting the effects of other members of the parental generation. Although sometimes intergenerational transmission is distinguished from genetically determined continuity and defined as the process through which an earlier generation psychologically influences attitudes, characteristics, and behavior (van Ijzendoorn, 1992), we consider intergenerational transmission to include both genetic and non-genetic pathways, recognizing that the two may be intertwined, leading to genotype-environment correlation.

## 3 Cultural mechanisms of intergenerational transmission

Several mechanisms of transmission of psychopathology and parenting behaviors have been proposed.

First, the shared environment of parents and children might affect the characteristics and behavior of both. This shared environment involves the broader family and home context, such as socioeconomic factors, household chaos, and cultural factors (Deater-Deckard, 2014). Living in the same neighborhood and in the same family house may constitute factors stimulating intergenerational continuity. Such cumulative continuity in individuals’ environment reinforces certain behaviors or interactional styles, thereby sustaining the behavior pattern across the life course and across generations (Caspi et al., 1989). For example, low SES might affect aggression in the parent and in the child. Thus, continuity across generations might be reinforced by sharing the same physical, social, economic, and cultural circumstances.

Second, parenting behavior can be transmitted across generations, but can also serve as a mechanism that explains transmission of psychopathology across generations (Conger et al., 2003; Serbin and Karp, 2004). Parenting is a broad and multifaceted construct that refers to how individuals raise their children, which also includes feelings and cognitions regarding child-rearing (O’Connor, 2002). Traditionally, two core aspects of parenting are distinguished: one pertaining warmth, responsiveness, or support, and the other including control or demandingness (e.g., Baumrind, 1968). Both aspects may include dimensions of parenting that are specific to certain situations (e.g., discipline strategies; Hallers‐Haalboom et al., 2016), or specific to stimulating certain skills (e.g., emotion and regulation; Eisenberg, 2020; Smetana, 2017). In line with social learning theory, transmission might occur through a process of modeling, in which children observe their parents’ behaviors and imitate those behaviors (Patterson, 1998). In line with attachment theory, experienced parenting is likely to influence children’s development and internal working models of relationships, which could then have consequences for their own behavior and relationships (Bowlby, 1988).

Cross-sectional and longitudinal studies show ample evidence for retrospective and prospective associations between parental characteristics and parenting behaviors, as well as between parenting and child behaviors, and this is often interpreted as evidence for the mediating role of parenting in intergenerational transmission (e.g., Bridgett et al., 2015). Prospective intergenerational research on these mechanisms is scarce. A prospective longitudinal study including data from grandparents, parent, and children found continuity in parental monitoring and harsh discipline across generations, but continuity in parenting practices did not mediate the intergenerational continuity in externalizing behavior (Bailey et al., 2009). Comparably, maternal and paternal involvement were found to be directly related to child self-control, but did not significantly mediate the effect of parental self-control on child self-control (Boutwell and Beaver, 2010). However, Dogan et al. (2007) found that adolescent-reported parenting mediated the longitudinal associations between adolescent-reported antisocial behavior of parents and adolescents. Also, a short-term longitudinal study (Ehrensaft et al., 2003) found a mediating role of parenting in mothers' history of onset of conduct disorder before age 15 and adolescent antisocial behavior. Thus, although parenting is often assumed to be a mediating mechanism in the intergeneration transmission of psychopathology, evidence so far is inconclusive.

Parenting not only can be looked at as a mediator in the process of intergenerational transmission, parenting behavior itself is thought to be transmitted from one generation to the next as well (Belsky, 1984). Associations of parenting across generations have particularly been reported for harsh and abusive parenting. The oldest observations of intergenerational transmission of parenting practices come from researchers and clinicians noting that parents who use abusive parenting practices often report to be abused themselves (Spinetta and Rigler, 1972). Most studies indeed report a modest association between maltreatment and having a history of maltreatment (see Thornberry et al., 2012; for a review; Widom et al., 2015). Several of these studies suffer from various methodological problems, and the studies with a weaker study design tend to report the strongest intergenerational effects. Many studies rely on non-representative samples, do not have a prospective design, and rely on single reporters (Thornberry et al., 2012; Van Ijzendoorn, 1992).

In recent years, a number of prospective studies have addressed the biases that come with retrospective reports of parenting (e.g., Belsky et al., 2005; Neppl et al., 2009; Kerr et al., 2009; Savelieva et al., 2017; Raby et al., 2015). Most of these studies have focused on the intergeneration transmission of harsh and abusive parenting, but intergenerational continuity has also been reported for constructive parenting. Chen and Kaplan (2001), for example, reported that experienced constructive parenting in adolescence was (weakly) related to self-reported constructive parenting two decades later. Part of this association was mediated, for example by quality of interpersonal relationships and psychological disturbance, but the remaining direct effect of experienced constructive parenting in adolescence on self-reported constructive parenting was interpreted as an indication of a role-specific modeling process.

Several socioeconomic circumstances, behaviors, and traits, including psychopathology, have been identified as mediators of continuity in parenting behavior across generations. The potential mechanisms of stability in parenting have been studied for both negative and positive parenting practices (see Kerr and Capaldi, 2019). Various prospective studies demonstrate that experiencing negative parenting practices contributes to the development of emotional and behavioral problems, such as antisocial behavior and depression, which in turn may increase the chance of repetition of these negative parenting practices in the next generation (e.g., Neppl et al., 2009; Scaramella et al., 2008; Thornberry et al., 2003; Rothenberg et al., 2017; but also see Bailey et al., 2009). The transmission of positive parenting practices, in contrast, have been found to be partially explained by educational success and social competence of G2 parents (e.g., Kerr et al., 2009; Neppl et al., 2009; Shaffer et al., 2009). For instance, a prospective longitudinal study from early adolescence into middle adulthood showed that perceived satisfying experiences with parents during early adolescence are positively related to marital satisfaction and educational attainment in young adulthood, which, in turn, are positively related to individuals’ use of constructive parenting in middle adulthood (Chen et al., 2008). In this case, marital satisfaction and educational attainment accounted for most of the direct effect of the intergenerational transmission of constructive parenting.

Two factors that might be particularly relevant in mediating the transmission of parenting behavior and psychopathology are self-regulation and social competence. Both self-regulation and social competence have longlasting effects on individual functioning in a variety of areas. Individuals who showed difficulties with self-control in preschool and early childhood were found to have poorer health, more substance abuse, more financial difficulties, higher delinquency, and lower quality of parenting in adulthood (e.g., Moffitt et al., 2011, 2013). Comparably, social competence in early childhood was related to young adult outcomes in multiple domains, such as education, employment, delinquency, substance use, and mental health (e.g., Jones et al., 2015). Evidence suggests that self-regulation is affected by parenting behavior, as parenting might mediate the intergenerational transmission of self-regulation (for a review, see Bridgett et al., 2015). Also, a prospective study of intergenerational continuity in parenting quality with assessments of G1 parenting and G2 social competence at each time point and assessment of G2 parenting at a 20-year follow-up found social competence to mediate continuity in parenting across generations (Shaffer et al., 2009).

These examples illustrate the complex transactional dynamics of parent and child characteristics and behaviors and parenting processes that together explain intergenerational transmission. Through a continuous process of reciprocal associations between parenting and psychopathology in both the first and second generation, parental psychopathology might elicit maladaptive parenting, and parenting is transmitted both directly through processes of modeling and internal working models and indirectly through its effects on offspring’s psychopathology.

Most of the research on non-genetic mechanisms of transmission did not take into account that such transmission may be confounded by genetic factors. Importantly, some studies with a genetically sensitive designs suggest that environmental transmission occurs after accounting for genetic processes. For example, children-of-twins studies showed significant direct environmental transmission from parents to their adolescent offspring for anxiety and neuroticism (Eley et al., 2015), for depression (for a review, see Natsuaki et al., 2014), and for antisocial behavior (Silberg et al., 2012).

## 4 Disentangling genetic and non-genetic transmission

Parents and children share a common genetic background, which accounts for part of the intergenerational transmission of characteristics and behavior. Children receive 50 % of their nuclear DNA from each biological parent (they receive all mitochondrial DNA from their mothers). Shared genes may affect child and parent behavior directly, resulting in similarities in behavior across generations. The shared genetic makeup of children and parents might also be correlated with the social environment they encounter, through gene-environment correlation (rGE). For instance, parents may transmit a genetic liability for antisocial behavior to the child and simultaneously provide an abusive rearing environment (Jaffee et al., 2004), or may transmit a genetic disposition for high behavioral control and create a tidy and organized home environment (Willems et al., 2019). This association between the genotype a child inherits from his or her parents and the environment in which the child is raised is referred to as passive gene–environment correlation (passive rGE). In this case, although the parenting environment might be associated with the child’s behavior, the association is in part caused by their genetic resemblance.

Children may also evoke certain parenting styles (evocative or reactive rGE; Kopala-Sibley et al., 2017; Avinun and Knafo, 2014), where the child’s genotype elicits behavior in their parents (e.g., particular parentings styles) or where the child actively seeks out particular exposures and experiences. For example, when offspring’s (G2) genotype is associated with their mother's (G1) parenting, this may indicate an evocative gene-environment correlation. Dobewall et al. (2019) found in a genome-wide association study that offspring genotype was associated with their mother's (G1) intolerance, but not with their own warmth and intolerance, suggesting that children elicit certain parenting behaviors based on their genetic makeup.

Further, active gene–environment correlation (active rGE) occurs when individuals select certain environments due to their genetic makeup. For example, adolescents’ genetically influenced personality characteristics have been found to affect adolescents’ selection of risky peer, family, and school environments, which subsequently affect substance use (Hicks et al., 2013). These examples of gene-environment correlations have in common that environmental influences explaining intergenerational transmission might in part reflect genetic effects.

The shared genetic makeup of parents and offspring may also moderate the influence of the social environment through gene-environment interactions (GxE). Gene–environment interactions could arise when environment only affects certain traits or characteristics if offspring possess specific genetic characteristics. Alternatively, the influence of genetic liability to develop certain behavior may depend upon exposure to specific environmental influences. Evidence for such GxE interaction has been suggested for example for depressive symptoms (review: Dunn et al., 2011): Individual differences in genetic makeup interact with exposure to maladaptive environments, such as chronic stress, stressful or negative life events, and maladaptive parenting, in predicting youth’s depressive symptoms. In the context of intergenerational transmission, Oxytocin Receptor (OXTR) genotype moderated the association between early exposure to maternal depression and depressive symptoms in adolescence (Thompson et al., 2014). Genes that have been suggested as influencing the development of various types of antisocial behavior are the dopamine transporter gene (DAT1) and the dopamine receptor genes DRD2, DRD3, DRD4, and DRD5, the serotonin transporter promoter polymorphism (5-HTTLPR) and a number of serotonin receptor genes, and genes that are implicated in the production of enzymes that metabolize neurotransmitters, such as the catechol-O-methyltransferase gene (COMT), and the monoamine oxidase A (MAOA) gene (Beaver et al., 2015), although many of these findings still await replication in well-powered genomewide association studies. One GxE interaction concerns the MAOA–maltreatment interaction: low-activity MAOA alleles were associated with antisocial behavior only in males who had been maltreated as children (MA: Kim-Cohen et al., 2006). Other examples of possible GXE interaction concern the interaction between 5HTTLPR and exposure to delinquent peers in the prediction of self-control (Beaver et al., 2015).

Recent genetic studies of candidate genes point to four systems underlying maternal behavior, that is, the dopamine, serotonin, and neuropeptide oxytocin and arginine vasopressin systems (for reviews, see Mileva-Seitz et al., 2016; Lomanowska et al., 2017). A number of genes involved in the functioning of the dopamine system have been related to differences in negative and sensitive parenting, in particular with mothering behavior. Similarly, associations of maternal behavior with gene variants coding for the serotonin transporter 5HTT, genes implicated in the functioning of oxytocin, and the gene for arginine vasopressin have been reported. In addition to direct effects of these genetic variants, GxE interactions have been reported for polymorphisms in these genetic systems, with early life adversity and quality of care that the mothers received from their own parents moderating the effect of the genetic variants on sensitive parenting (for a review see Lomanowska et al., 2017). Also, in a three-generation study, the oxytocin system was found to interact with experienced parenting to affect bonding across all three generations (Fujiwara et al., 2019): Mothers who reported high overprotective parenting from grandmothers showed more rejection toward their infants and had lower oxytocin levels only when they were OXTRrs53576 G carriers (AG/GG). Comparably, grandmothers who reported higher overprotection from great-grandmothers showed poorer parenting style only when they were OXTRrs2254298 GG carriers. Infants whose mothers reported more rejection towards the infant had higher oxytocin levels only when they were OXTRrs2254298 A carriers (AA/AG). As above, we note that many candidate gene and candidate interaction studies await replication in genomewide, well-powered research projects. Duncan and Keller (2011) in a review of candidate gene-by-interaction studies concluded that “cG × E studies are underpowered. Low power along with the likely low prior probability of a given cG × E hypothesis being true suggests that most or even all positive cG × E findings represent type I errors”.

## 5 Epigenetic processes

In addition to the effects of genetic and cultural transmission, epigenetic processes are increasingly considered as a molecular mechanism involved in intergenerational transmission. The expression of genes is in part regulated by epigenetic inﬂuences and environmental exposure can alter functional genetic expression, without altering the underlying genetic sequence, and as such affect behavior (Ng et al., 2012). Although studies on the role of epigenetic processes so far have often focused on physiological functioning and risk for disease (Relton and Davey Smith, 2012), epigenetics is also an important mechanism by which environmental factors can affect psychosocial functioning (Barker et al., 2018; Mill and Petronis, 2007). Changes in gene expression may occur as a result of for instance exposure to prenatal maternal smoking, environmental stress or major emotional trauma (Meaney and Szyf, 2005). Examining epigenetic processes in longitudinal cohort studies can reveal causal processes that may remain unrevealed when focusing on genetic variation and can clarify how gene-environment interactions contribute to development and intergenerational transmission (Yehuda and Bierer, 2009; Meaney, 2010; Moore et al., 2019). Because of its feasibility of application in large numbers of samples, the best studied indicator of the epigenetic processes is DNA methylation, or the chemical coating of the chromosomes that regulates the transcription of genes. DNA methylation concerns the process in which a methyl group (CH3 group) is added to cytosine in a cytosine-phosphate-guanine (CpG) group within a gene-promoter region. This change in the structure of the DNA results in changes in gene transcription (Meaney, 2010; Mileva-Seitz et al., 2016). Whereas hypomethylation of regulatory sequences is related to increased gene expression, increased methylation is related to transcriptional suppression. By examining changes in gene-specific methylation over time and relating these changes in methylation to changes in the psychosocial environment, it is not only possible to examine whether and how the epigenome changes over time as a result of important environmental changes in children’s lives, but also whether these epigenetic processes are involved in mediating the association between genetic or environmental risk factors and psychosocial adjustment. For example, DNA methylation levels are susceptible to exposure to life adversities such as exposure to child abuse (Van der Knaap et al., 2014), and play a role in modifying the hypothalamic-pituitary-axis (HPA) response (Van der Knaap et al., 2015), which affects adaptation to the environment (Kofink et al., 2013). In addition, individual differences in methylation levels may also be partially explained by genetic differences (van Dongen et al., 2016, 2018). Animal studies, and to a much more limited extent human studies, have shown that early adversity, including socioeconomic disadvantage and maternal care, affects gene expression and neural function, thereby contributing to intergenerational transmission (for reviews see Meaney, 2010; Scorza et al., 2019). These studies show how environmental factors, including parent–offspring interactions and social-economic disadvantage, affect epigenetic mechanisms as well as own (parenting) behavior For example, mothers’ experience of early interpersonal violence and maltreatment was found to be related to methylation of the promoter region of the glucocorticoid receptor gene NR3C1, which in turn related to neural response to interactions with their children (Schechter et al., 2015).

Moreover, epigenetic changes are heritable themselves (Meaney and Szyf, 2005). Through complex physiological processes, parents can not only transmit genetic characteristics to their children but also epigenetic characteristics, especially if these epigenetic characteristics are based on very stressful and life-threatening experiences. As noted, most studies investigating epigenetic intergenerational transmission are based on animal models, leaving it unclear whether intergenerational epigenetic inheritance exists in humans (van Otterdijk and Michels, 2016; Scorza et al., 2019). A few studies suggest that epigenetic transmission might also occur among humans. For instance, a cross-sectional study of 22 holocaust survivors and their (adult) children found that holocaust exposure was related to cytosine methylation within a gene encoding for glucocorticoid receptor sensitivity (Yehuda et al., 2016). A study of children and grandchildren of individuals who survived the Dutch famine of 1944–45 found persistent changes in DNA methylation in those with prenatal famine exposure compared to those not exposed prenatally to the famine (Tobi et al., 2009). Although these studies were not able to differentiate between epigenetic inheritance, selection processes or social transmission, they provide some evidence that stress associated with early adversity could affect future generations, independent of and in interaction with those future generations’ life experiences (Meaney, 2010; Scorza et al., 2019).

Overall, research into the epigenetic mechanisms in intergenerational transmission among humans is in its early stages. Lomanowska et al. (2017) proposed a model in which main effects of genes and environment, interactions between genes and environment, and mediating effects of epigenetics together shape parenting. In their model, parental genes might be associated with the parental environment through passive and active gene–environment correlations. In addition, grandparenting might affect the parental epigenome, which subsequently affects parental behavior. Changes in methylation patterns due to grandparenting behavior might result in altered functioning of the oxytocin, serotonin, and cortisol system, which might elicit insensitive parenting. Parental susceptibility genes, inherited from the grandparents, may moderate this mediation as well as the inﬂuence of environmental factors on parental behavior. Longitudinal studies are now needed to further explore these mechanisms in humans, thereby taking into account both epigenetic processes and current social and contextual factors (Scorza et al., 2019).

## 6 Moderators of intergenerational transmission: situational factors and child characteristics

Notwithstanding the evidence for cultural transmission of psychopathology and parenting behavior, cultural transmission tends to be weak or moderate when compared to the effects of genetic transmission. Intergenerational transmission may not occur in all families, and some children seem more strongly affected by the behavior or characteristics of their parents than others. It is therefore important to not only focus on the underlying mechanisms of transmission of characteristics and behavior from one generation to the next, but also to understand discontinuities across generations (Rutter, 1998). Research has therefore also focused on understanding the factors that may increase or decrease the chance that intergenerational transmission takes place (Belsky et al., 2009; Conger et al., 2009).

Among the conditions that increase the chance of transmission from one generation to another are situational characteristics such as low socioeconomic status (Goodman et al., 2011). Aspects of the parent’s relationship with their partner, such as marital status, relationship quality, and partner violence have also been found to moderate intergenerational transmission of depression, externalizing behavior, and parenting (e.g., Goodman, 2020; Jeon and Neppl, 2019). In addition, the quality of the offspring’s partner relationship moderates intergenerational transmission (e.g., Belsky et al., 2005; Conger et al., 2013). Interestingly, there is also evidence for the continuity in quality of partner relationships across generations, yet those offspring that have high quality partner relationships despite their parents’ negative marital quality might be able to break the intergenerational transmission cycle and show positive parenting or behavior. A meta-analysis revealed that certain types of safe stable nurturing relationships between parents and other adults, such as a romantic partner or co-parent, or adult social support resource, decreased the continuity of maltreatment (Schofield et al., 2013).

Among child characteristics, a potential moderator of intergenerational continuity is gender, as some studies found stronger transmission of parenting and psychopathology for daughters than for sons (Belsky et al., 2005; Besemer et al., 2017; Thornberry et al., 2003). However, this moderating effect of gender is not always found (e.g., Goodman, 2020; Neppl et al., 2009; Shaffer et al., 2009), and some studies even report stronger continuity for boys than for girls in parenting (Savelieva et al., 2017). Child age also has been identified as a moderator, with stronger effects for younger children (Goodman, 2020). Child temperament (Wang and Li Lui, 2018), emotion regulation, and stress-reactivity (Scaramella and Conger, 2003) also have been reported to moderate intergenerational transmission of corporal punishment and hostile parenting. These results suggest that child temperament conditions intergenerational continuity in parent hostility, yet they might also indicate differential susceptibility in a way that individuals sensitive to environmental influences will be adversely affected by negative environments and benefit from supportive ones (Ellis et al., 2011). Intergenerational transmission might thus be stronger for those individuals susceptible to environmental influences.

In sum, intergenerational transmission takes place due to a complex interplay between genetic and non-genetic factors. Despite substantial evidence for mediating processes that explain intergenerational transmission and moderating factors that strengthen or weaken intergenerational transmission, many studies on intergenerational transmission are hampered by methodological limitations. Next, we discuss some methodological advances and considerations in the study of intergenerational transmission.

## 7 Methodological considerations in intergenerational transmission

There are various methodological components that make well-designed cohort studies promising candidates to examine intergenerational transmission. These methodological components have been described in multiple reviews (e.g. Dubow et al., 2003; Thornberry et al., 2012; Thornberry, 2016; Kerr and Capaldi, 2019). There are various variations in cohort studies that extend their power to examine intergeneration transmission, such as reliance on a representative sample, the assessment of confounding variables and broad contextual data, and assessment of multiple children in the same family (Dubow et al., 2003; Kerr and Capaldi, 2019; Thornberry et al., 2012; Thornberry, 2016). Building on previous reviews, we discuss various methodological considerations for studies on intergenerational transmission.

### 7.1 The need for prospective longitudinal designs

Many studies on intergenerational transmission use cross-sectional data, either with concurrent assessments of parental and child behavior at one time point, or with assessments of perceptions of their own parenting styles or behavior and retrospective recollections of the parenting styles or behavior of their parents. In recent years, the number of prospective and longitudinal studies spanning two or three generations of children has increased. This is important, as intergenerational transmission assumes a temporal ordering, for which longitudinal data are required (Cairns et al., 1998). Repeated measures during G2 development allow assessment of multiple, often cascading, developmental pathways that may explain intergenerational similarities (Thornberry, 2016). Moreover, prospective longitudinal studies avoid bias due to retrospective reports (Hardt and Rutter, 2004). In this section we discuss several other considerations for using prospective longitudinal designs to study intergenerational transmission.

### 7.2 Intergenerational transmission depends on age

The extent to which behaviors or characteristics are transmitted across generations might vary depending on the age at which these behaviors and characteristics are assessed (Thompson, 2014). Some behaviors might not manifest themselves immediately, but only at later ages. For example, health related conditions might have a low prevalence rate in early childhood and emerge only later in the life cycle (Thompson, 2014). Also, transmission of income might show different estimates at different points in the life cycle. For these kind of characteristics, intergenerational transmission should ideally be examined when individuals from both generations have roughly the same age.

Other characteristics might emerge at earlier ages, but might reflect developmental heterogeneity. That is, these characteristics might manifest in different forms or with different symptoms at different ages. For instance, intergenerational transmission of aggressive behavior might show at an early age, yet whereas aggression in early childhood might be characterized by hitting and biting, adult aggressive behavior might be characterized by verbal aggression or serious abuse. The degree to which differing behaviors are assessed across generations likely attenuates the effect size of the intergenerational continuity. Moreover, also the dyadic and intergenerational transmission processes might change as a function of development of both individuals and their relationships (Dubow et al., 2003).

Thus, to examine intergenerational transmission, long-term prospective studies are needed that follow different generations within families over the life course with age-appropriate measures of the same behaviors or characteristics under study, and measurements within narrow age ranges, which allows to examine changes in the strength of intergenerational transmission as children, and parents, get older (Thornberry, 2016). A wide variability in age of G3 children can be quite common in three-generation studies when assessments occur according to calendar year, instead of age (e.g., Shaffer et al., 2009). Ideally, studies should be designed to examine intergenerational similarity of the behavior of interest in the same developmental stage (Thornberry, 2016). This requires long follow-up measurements. For instance, intergenerational transmission of parenting requires two generations of parents. If parenting is assessed in adolescence, it will take another 10–15 years before a large number of these adolescents will be parents themselves. Furthermore, it will take even longer (i.e., 25–30 years) before a substantial percentage of these former adolescents are parents of adolescents.

### 7.3 Accounting for potential child effects

Empirical studies have shown the importance of including the effects of child behavior on subsequent parenting (Meeus, 2016). This limitation might also apply to genetically sensitive designs such as the children-of-twins design, in which the effect of parenting behavior is assumed to affect child outcomes, and not the other way around (reactive gene-environment correlation), which in some cases might not be justified. A study applying a twin- and an adoption study revealed that associations between parental depressive symptoms and offspring internalizing and externalizing problems remain after accounting for shared genes, and that child-to-parent effects were also present (McAdams et al., 2015). Longitudinal studies are needed to disentangle the reciprocal effects of parenting and child behavior (Dubow et al., 2003). Although longitudinal studies may not be optimally suited to address causal inferences, researchers can examine how child behavior predicts parent behavior, whilst controlling for prior parent behavior, and vice versa: how parent behavior predicts child behavior, whilst controlling for prior child behavior. In this respect, following multiple children from one family within the same generation (i.e., twins or siblings) can be beneficial to guide causal explorations and examine differential associations across generations (Kerr and Capaldi, 2019).

### 7.4 Distinguishing characteristics of the parental generation or offspring rearing experiences

The behavior of the parental generation might affect behavior of their offspring differently depending on when parent behavior was assessed. Continuities driven by the characteristics of the parental generation and continuities driven by the experiences of rearing provided for the offspring may not necessarily be the same (Rutter, 1998). For example, children were at an increased risk of becoming teenage mothers if their mothers were teenagers at the time their first child was born (Hardy et al., 1998), suggesting that age of parents at birth might be less important for intergenerational continuity of teenage parenthood than age of parent at birth of the first child in the family. Comparably, using a sibling comparison design, concurrent but not perinatal maternal depression was significantly associated with offspring internalizing and externalizing problems during preschool years (Gjerde et al., 2017), and concurrent but not perinatal maternal anxiety was significantly associated with offspring internalizing problems (Gjerde et al., 2020). Moreover, the effect of concurrent maternal depression on internalizing problems increased with child age. In a review on intergenerational transmission of depression, Goodman (2020) stressed that not only the timing of maternal depression, but also the severity, chronicity, and comorbidity might affect the extent of transmission.

### 7.5 Including fathers and mothers in the study of intergenerational transmission

While most intergenerational transmission studies have included one individual per generation and thus focus on the influence of one parent, children are affected by and share genes with both parents. Ideally, studies of intergenerational transmission should therefore include both parents’ behavior or parenting (Thornberry, 2016). Composite parenting scores from both parents have been found to relate more strongly to child behavior than parenting scores of a single parent (Dubow et al., 2003). Including fathers and mothers also allows to test whether cross-generational continuities are stronger for same-sex parent–child dyads than mixed-sex parent–child dyads. Relatedly, parenting measures based on various methods and reporters allow to test the robustness of associations, although they also complicate the interpretation of intergenerational continuity by risking “comparing apples to oranges” (Kerr and Capaldi, 2019).

Although several studies do include measures of both parents of Generation 1 as adults, including both Generation 1 parents during their own childhood constitutes a major challenge for research. That is, samples need to be extremely large and inclusive to have sufficient participants followed from childhood onwards that select their partner within the sample (Rutter, 1998). To be able to include data on childhood parenting experiences of both parents, some retrospective data will therefore likely be inevitable.

Including both parents is even more important in the study of intergenerational transmission because of processes of assortative mating (Maes et al., 1998). Parents tend to select partners that are similar to themselves, due to social homogamy, phenotypic assortment or to similarity-attraction processes. When both parents share the behavior or parenting style, they might reinforce these behaviors in each other, thereby exposing children to these behaviors even more. In addition, children might have a greater likelihood of having a genetic propensity for the behavior in question. Thus, environmental risk factors and genetic risk factors will tend to covary in such a way that they increase the odds for the offspring to develop the behavior in question. Thus, due to assortative mating patterns, in certain families risk factors tend to co-occur (Boutwell and Beaver, 2010), and without including both parents of Generation 1, these processes of intergenerational transmission cannot be disentangled.

### 7.6 Genetic designs

Genetically sensitive designs can provide a useful tool to get more insight into the processes that are involved in intergenerational transmission and to disentangle genetic and non-genetic influences. One such design is the classical twin study (Martin et al., 1997; Boomsma et al., 2002). Here a comparison in trait resemblance is made between identical or monozygotic (MZ) twins, who share (almost) all of their segregating alleles, and fraternal or dizygotic (DZ) twins, who are on average 50 % genetically related. The difference in genetic relatedness between these two types of twins can be employed to attribute individual differences between children to additive genetic (A), non-additive or dominant genetic (D) influences, to common environment (C) representing the shared (by the twins who grow up in the same family) environment and non-shared environmental (E) effects (which also includes measurement error; Posthuma et al., 2003). There is abundant evidence that most environmental effects that influence behavior are not shared by children growing up in the same family (Plomin, 2011). Although this might seem to suggest that parental traits and parenting play no role in children’s development, it can also indicate that the most important aspects of the home environment that influence a child are specific for each child within a family. Twin studies can determine the extent to which genes and environment explain individual differences in a trait, but cannot identify the specific genes or the aspects of the home environment that are important.

A children-of-twin study provides the opportunity to disentangle the effect of specific parental characteristics on children’s behavior into genetic and non-genetic transmission, while taking gene-environment correlation into account. Parents and their offspring share 50 % of their genes. The logic underlying a children-of-twin study is that offspring of MZ twins are as genetically related to the co-twin of their parent (their uncle/aunt) as they are to their own parent while this is not the case for offspring of DZ twins, who share 25 % of their genes with the co-twin of their parent. All children share a home environment with their parent and (generally) not with their aunt/uncle (McAdams et al., 2014). Comparing the resemblance between parents and offspring (parent-offspring correlation) with the resemblance between aunts/uncles and nieces/nephews (avuncular correlation) in MZ and DZ twin families gives information on the underlying mechanisms of intergenerational transmission. A higher parent-offspring correlation than the avuncular correlation would indicate cultural transmission. A higher MZ avuncular correlation than the DZ avuncular correlation indicates genetic transmission. One limitation of a children-of-twins study is that even though it controls for familial confounding, the association could still be due to confounding by other parent or child characteristics not included in the study.

A polygenic score (PGS) study employs an individual’s genetic predisposition for a trait based on his/her measured genotype, which is inherited from both parents. In genome-wide association studies (GWAS) the effects of millions of genetic variants on a large range of human traits have already been estimated. For each individual, the effects of (a subset of) these genetic variants can be summarized in a PGS by taking the sum of the number of effect alleles present at each locus weighted by their effect size as estimated in a specific GWAS (Purcell et al., 2009). If, in an independent sample, a PGS for a certain trait predicts an outcome in childhood this would indicate that there is genetic transmission from parents to offspring development. Studies with smaller sample sizes could also rely on candidate gene approaches (Thornberry, 2016).

In families in which two generations are genotyped, that is, mother/father and their offspring, we can extend the PGS study to two PGSs, one based on alleles transmitted from parent to their offspring and one based on non-transmitted alleles, because parents transmit only one of their alleles at each autosomal locus to the next generation (Kong et al., 2018; Bates et al., 2018). If the non-transmitted alleles affect an outcome in the offspring, this indicates genetic nurturing: the home-environment is influenced by the parental genotypes, which in turn affects children’s development. The effect of the non-transmitted PGS on childhood outcomes includes only genetic nurturing effects while the transmitted PGS includes both direct genetic and genetic nurturing effects. Studies applying such a “virtual-parent method” showed that children with higher polygenic risk scores on educational attainment scored higher on educational attainment, and that, in addition, parents’ non-transmitted polygenic risk scores were related to child educational attainment (Bates et al., 2018; Kong et al., 2018), which was mediated by parental SES (Bates et al., 2018). In the Netherlands, we assessed the effects of transmitted and non-transmitted PGS in adults and in 12-year old children for educational attainment (EA) and ADHD. In adult offspring, both the transmitted and non-transmitted EA PGSs were associated with offspring EA, providing evidence for genetic nurturing. In children, EA was associated with the transmitted EA PGSs, but not with the non-transmitted EA PGSs. ADHD symptoms in children were associated with transmitted EA and ADHD PGSs (de Zeeuw et al., 2020). This study also illustrates our points about the importance of considering the role of age in transmission research. One current limitation of (non-)transmitted PGS studies is that the investigation of parental behavior and parenting is limited to traits such as educational attainment, ADHD, BMI, cardiovascular risk and some other somatic diseases and psychiatric disorders (Chang et al., 2018), for which a large enough GWAS is available, so that weights for PGS can be reliably obtained.

Analogous to GWAS, epigenetic-wide association studies (EWAS) can estimate the effects of epigenetic variants on human traits. The focus typically lies on characterizing genome-wide DNA methylation variation, but additional epigenetic marks may also be examined (Tsai et al., 2012). Moreover, as some epigenetic changes are heritable themselves (Meaney and Szyf, 2005; van Dongen et al., 2016), EWAS across multiple generations in human subjects can provide valuable insights in studies on intergenerational transmission.

## 8 The four cohort studies in the Consortium on Individual Development

Even though multimethod repeated measures have been the standard in the study of intergenerational transmission since decades (Rutter, 1998), long-term cohort studies that follow individuals within families across multiple generations are still relatively scarce. In Work Package 3 of the Consortium on Individual Development, four large prospective longitudinal cohorts are included that track development across several generations. Each of the cohorts has its own strengths in studying intergenerational transmission, and together, they allow a better understanding of the genetic and non-genetic mechanisms underlying transmission from one generation to the next. Two cohorts with two- and three-generation data will be used to study the epigenetics of cross-generational transmission. In addition, two ongoing longitudinal multi-informant three-generation cohorts with comparable design and measures will be included to allow testing effects from grandparents to parents to children over time. Both studies are currently being enriched with measures of the third generation and with epigenetic data. Together, the prospective longitudinal cohorts are able to provide much needed information on the underlying mechanisms of intergenerational transmission as well as on which conditions moderate the transmission process and explaining why quality of parenting and behavior in one generation are transmitted to the next (Belsky et al., 2009). In Table 1, we summarized the methodological considerations for studies on intergenerational transmission, and demonstrate how the four cohorts take these considerations into account.

**Table 1:** Measures and methodological considerations and the four CID cohorts.

	NTR	Generation R	RADAR	TRAILS
**General considerations**				
Representative sample	x			x
Assessments of confounding variables	x	x	x	x
Longitudinal design	x	x	x	x
Repeated measures during G2 development	x	x	x	x
During G1 pregnancy				
During G2 early childhood	x			
During G2 middle childhood	x			
During G2 adolescence	x		x	x
During G2 early adulthood	x	x	x	x
During G2 middle adulthood	x	x		
Repeated measures during G3 development	v	x	x	x
During G2 pregnancy		x		x
During G3 early childhood	v	x	x	x
During G3 middle childhood		x		
During G3 adolescence		x		
During G3 early adulthood				
Same concept across generations	x	x	x	x
Self-regulation	x	x	x	x
Social competence	x	x	x	x
Psychopathology	x	x	x	x
Parenting		x	x	x
G3 measurements at narrow ages	v	v	x	x
Assessment of multiple children in the same family	x	x	x	x
Multiple G2 children	x	Rarely	x	Rarely
Multiple G3 children		x		x

**(Epi-) Genetic and biomarker data**				
Twin study	v			
Children of Twins study	v			
GWAS across multiple generations	v			x
GWAS G1	v			x
GWAS G2	v		x	x
GWAS G3	v	v		
EWAS across multiple generations	v			
EWAS G1	v			
EWAS G2	v		x	
EWAS G3		v		
Candidate genes across multiple generations	v			
Candidate genes G1	v			
Candidate genes G2	v		x	x
Candidate genes G3				
Biomarkers (e.g. metabolomics, EEG, MRI)	v			
Biomarkers G1	v			
Biomarkers G2	v		v	x
Biomarkers G3	Rarely	x		

**Cultural processes**				
Broader contextual data (neighborhoods, SES etc.)	x	x	x	x
Inclusion of fathers of mothers	x	x	x	x
G1 mothers	x	g, v	x	x
G1 fathers	x	g, v	x	x
G2 fathers	v	x	x	x
G2 mothers	v	x	x	x
Multiple measures of parenting			x	x
Multiple methods (e.g., questionnaire and observations)		x	x	x
Multiple reporters		x	x	x

*Note*. v = limited to subgroups. g = only included as grandparents.

### 8.1 Netherlands Twin Register

The Netherlands Twin Register (NTR; www.tweelingenregister.vu.nl) enlists twins, higher-order multiples and their family members nation-wide without any exclusion criteria. Since the early 1980s, NTR has enrolled around 120,000 twins and a roughly equal number of their relatives. The majority of twin families take part in longitudinal survey studies, and subgroups take part in biomaterial collection, e.g., DNA isolation and biomarker studies and in dedicated projects for example neuropsychological, EEG/MRI, and behavioral and cognitive traits. The resources and databases have been described in a series of papers (e.g., Boomsma et al., 2008; van Beijsterveldt et al., 2013). Starting around 1986, NTR systematically began to invite parents of newborn twins into the Young NTR (YNTR) with the help of a commercial ‘birth congratulation’ service. Additional recruitment of newborns and their parents is done in collaboration with the Dutch Society of Parents of Multiples (“Nederlandse Vereniging van Ouders van Meerlingen”: NVOM).

After parents return an informed consent form, mothers receive a first survey with questions on pre- and perinatal factors. After the twins’ second birthday, a survey on growth, health, developmental, and motor milestones is sent. At ages 3, 7, 9/10, and 12, both parents receive a survey, including the Child Behavior Checklist and the Conners’ Parent Rating Scale-Revised. At age 5, the parental survey includes a short version of the Devereux Child Behavior rating scale, the Child Behavior Questionnaire, and questions about health, day care, and additional sibs. At ages 7, 9/10, and 12, parents are asked for permission to approach the primary school teachers of twins and additional siblings in the family. Surveys also include questions regarding growth, parental characteristics and SES, and school performance. The survey sent to teachers includes the Teacher Report Form, the Conners’ Teacher Rating Scale-Revised, and the Social Skills Rating System. Academic achievement tests administered by Dutch primary schools are collected from teachers as well, including standardized tests in grade 1–6 (ages 6–12) from the Pupil Monitoring System (“Leerling Volgsysteem toetsen”) and the CITO test which is administered nation-wide in the last grade of primary school around age 12. After age 14, twins and their sibs are invited to provide self-reports, including the Youth Self Report and questions regarding personality, lifestyle, well-being, health-related traits and school performance. In YNTR, we also collected a reference panel for phenotype data to harmonize multiple measures of childhood behavioral problems in school-aged children. Throughout 2016, the Child Behavior Checklist, the Strengths and Difficulties Questionnaire plus a selection of A-TAC items (Autism-Tics, ADHD, and other Comorbidities inventory) were completed by both parents of twins born between September 2005 and October 2008.

Twin families with adolescent and adult twins were mainly recruited through city councils in the Netherlands, starting in 1985 with twin families in and around Amsterdam. In 1990–1993 NTR asked all city councils for permission to approach twin families. This created the Adult NTR, with the first surveys collected in 1991. Other means of recruitment are the NTR newsletters, the website, and events organized by the Dutch Twin Society. In total, 13 surveys have been sent to ANTR participants about every two to three years since 1991. Detailed information on phenotyping in adults and on non-survey based phenotyping in all age groups may be found in Ligthart et al. (2019).

Over the years, a total of 280,569 participants, including 59,520 complete twin pairs and 871 complete sets of triplets were registered at the NTR, 231,088 of whom are still contactable. The register includes 255,785 members of twin families and 24,784 teachers of children. Until now, the NTR has collected data on 70 % of all registered twin-family participants (i.e. excluding teachers). The NTR mostly includes two-generation extended families, i.e. twins, their siblings and their parents. The first infant twins who were enrolled by their parents are now becoming parents themselves and NTR has collected data on children from this third generation (*N* = 81). Other interesting groups with three-generation data include families where at least one of the parents and their offspring are twins (*N* = 730), as well as families where two siblings are both parents of twins (*N* = 469).

DNA collection (from peripheral blood samples or by buccal swabs) constituted an important element of multiple projects and continues for participants with rich phenotype information. Uniquely, nearly all DNA collection is in two-generation families. Genotyping is done for zygosity assessment in twins and for genome-wide association studies (GWAS). DNA samples are also increasingly analyzed for biomarker studies, e.g., in epigenetic projects. Other biomarkers in children include metabolomics data assessed in urine across platforms for amines, organic acids and steroid hormones.

### 8.2 The Generation R Study

The Generation R Study is a prospective population-based cohort from fetal life onward in a multi-ethnic urban population. All pregnant women (expected delivery date April 2002–January 2006) living in Rotterdam, the Netherlands, were invited to participate by their obstetrician or midwife during routine visits. In total, 9778 mothers were enrolled in the study, which was a response rate of 61 %. At age 1.5, 3, 6 and 10 years, children and their primary caregiver were invited to complete questionnaires and/or visit the research center (Kooijman et al., 2016). Questionnaire data available for this cohort of children and their parents covers, amongst others, measures of psychopathology, well-being, cognitive development, executive functioning, friendships, bullying, sleep, home environment, life events, feeding practices, substance abuse, and parenting styles. Observed data includes – but is not limited to – measures of cognitive development (four subtests of the Wechsler Intelligence Scale for Children V- WISC V), neurodevelopment (prenatal ultrasounds, neonatal head circumference and two waves of magnetic resonance imaging [MRI], the first wave: 6-to-9 year-old children, the second wave: 10 year-old children), bullying, social network, motor development (Touwen’s Neurodevelopmental Examination), executive functioning (NEPSY), observed parent-child interaction, risk-taking, sleep (actigraphy and sleep diaries), home environment observation (HOME) and a life events interview.

Next to questionnaire and observed data, DNA from children was extracted and used for genotyping using taqman analyses for individual genetic variants and using a genome-wide association scan (GWAS) using the Illumina 670 K platform in the children (*N* = 5732). DNA methylation (EWAS) is available in a subgroup of Dutch children on three different ages, at birth (*n* = 1339), at age 6 years (*n* = 493), and at age 10 years (*n* = 465). Samples were processed with the Illumina Infinium Human Methylation 450 BeadChip (Illumina Inc., San Diego, USA), which analyses methylation at 485,577 CpG sites. This subgroup is ethnically homogeneous, all from Caucasian decent, to exclude confounding or effect modification by ethnicity.

In a different randomly assigned subgroup of Dutch pregnant women (*N* = 1232) and their children, detailed assessments were conducted including, observations of parent-child interaction and behavior, infant-parent attachment, moral development, parental sensitivity observations, postnatal cranial ultrasound and prenatal psychiatric interviews asking about parental and grandparental psychopathology. Grandparents (G1 parents) are also a part of this study for a subgroup of the participants.

The assessment wave at 13 years is completed in autumn 2019. Preparations for the next wave, at age 17 years, have started. The next wave will include repeated measures of the questionnaires listed above. In addition a face-to-face semi-structured psychiatric interview (The Kiddie Schedule for Affective Disorders and Schizophrenia; K-SADS COMP, Kaufman et al., 2017) will be administered generating dimensional and categorical psychopathology outcomes according to DSM 5.

### 8.3 The RADAR study

The Research on Adolescent Development and Relationships (RADAR, Branje and Meeus, 2018; www.uu.nl/en/research/radar) cohort study is an ongoing Dutch longitudinal study that examines development from adolescence at age 12 into young adulthood at age 30. RADAR is a multi-method, multi-informant two-cohort longitudinal study. In addition to development of target participants during adolescence and young adulthood, RADAR also examines development of their parents, siblings, friends, and partners. In young adulthood, the children of the target participants of both cohorts and of their siblings are also included in the so-called “Generation 3” study. In total, 730 target adolescents participate. RADAR consists of two cohorts: RADAR Young (*N* = 497, 283 boys), 12 years old at Time 1 (2005) and RADAR Old (*N* = 233, 107 boys), 12 years old at Time 1 (2001). Attrition rates are very low as over 85 % of the original sample is still participating. Currently, the 10th wave of RADAR Young and the 13th wave of RADAR Old have been conducted. At least one more biennial measurement wave is foreseen. Thus, for RADAR Young data will be available between age 12 and 28, and for RADAR Old, data will be available between age 12 and 30.

The project includes self-report assessments of target adolescents as well as assessments of their families, best friends, and (in later measurement waves) intimate partners and their children. In the first 6 waves, RADAR includes annual multi-informant measures, among which parent reports on their own marital relationship, adolescent-, sibling-, and parent-reports of parent-child relationship quality and parenting, self- and other-reports of internalizing and externalizing problem behavior, and self- and other-reports of parent and adolescent personality, identity, social competence and self-regulation. In addition to questionnaire data, during the first 5 waves, adolescents and parents also participated in observed interaction tasks, as well as in measurement burst assessments of relational experiences and mood. Moreover, around age 17 adolescents participated in a lab visit, in which they conducted several neurocognitive tasks, among which a stop signal task, the IOWA gambling task, and a public speaking task.

From Wave 7 onwards intimate partners participate in the study and data are being collected every 2 years. In addition to measures on relationships with parents, personality and psychosocial adjustment, target adolescents also report about romantic relationship quality Genome-wide data of target participants in RADAR Young have been collected as well, when targets were about 17 and 26 years of age, and both genome-wide association studies (GWAS) and repeated epigenome-wide association studies data are available. Additional assessments of DNA will allow analyzing changes in gene-specific methylation over time within the same individual both from early to middle adolescence and from middle adolescence to early adulthood and relating these changes in methylation to changes in the psychosocial environment. Moreover, we can also examine whether these epigenetic processes are involved in mediating or moderating the association between genetic and/or environmental risk factors and development of identity and autonomy.

Since 2013, the target and sibling participants in RADAR Young and Old who have children are invited to participate in additional data collection centering around RADAR-G3: the next generation. Three waves of data collection are currently conducted. The participant and his/her partner fill out questionnaires about their child’s temperament, developmental milestones, psychosocial adjustment, and child-rearing when the 3rd generation child is 3 months, 24 months and 54 months old. In addition, a home visit is conducted when the child is 2.5 years old, with parent-child interaction tasks and child social competence and self-regulation tasks, and a school assessment takes place at age 5.

### 8.4 TRacking Adolescents’ Individual Lives survey

The TRacking Adolescents' Individual Lives Survey (TRAILS; http://www.trails.nl) is an ongoing multi-wave and multi-informant cohort study with bi- or triennial follow-up assessments that follows youth from preadolescence at age 11 into adulthood (Huisman et al., 2008; Oldehinkel et al., 2015; Ormel et al., 2012). The TRAILS target sample comprised of young adolescents from five municipalities in the north of the Netherlands, including both urban and rural areas. TRAILS consists of two prospective cohort studies, a population-based (*N* = 2,230) and a clinical (*N* = 543) cohort. The population cohort includes participants born between 1 October 1989 and 30 September 1991, who lived in the North of The Netherlands at the time of the baseline assessment in 2001/02. The clinical cohort consists of individuals from the same geographical region who were referred to a child psychiatric outpatient clinic in the Northern Netherlands any time before the age of 11. Data collection in this cohort started a few years later in 2004. To date, the population cohort has been assessed six times over a period of 15 years, with retention rates ranging between 72 % and 96 %. The clinical cohort has been assessed five times over a period of 11 years, with retention rates ranging between 74 % and 85 %. Currently, the seventh wave of TRAILS population cohort is being conducted and the sixth wave of TRAILS clinical cohort is imminent. After these data collection waves, data off the TRAILS population cohort will cover the period between ages 11 and 29 years, and data off the TRAILS clinical cohort the period between ages 11 and 25 years.

The research protocols of the TRAILS population and clinical cohorts are virtually identical for maximal comparability. The study collects data from multiple sources: in addition to self- and parent-reports (all waves) the database contains teacher-reported data (waves 1–3), sibling data (wave 3), peer nominations (waves 1 and 2), partner-reports (from wave 4 onwards) and registry-based data from preventive child healthcare and mental healthcare providers. Through questionnaires, interviews, blood and saliva samples, and tests, detailed information has been obtained on mental and physical health and well-being, physical development and physiological functioning, life events and difficulties, temperament/personality, self-perception, neurocognitive functioning, academic performance, social behavior, lifestyle, family characteristics, family functioning, peer and romantic relationships, work-related factors, sleep, religiosity, and genetic (genome wide) and epigenetic (candidate genes) make-up.

Since 2015, TRAILS participants and their partners have been invited to join in the add-on study TRAILS – The Next generation (TRAILS-Next) when they are expecting a child. For TRAILS-Next at least five waves of data collection are currently planned. The TRAILS participants and their partners enter the study during pregnancy, and are visited by research assistants at 3, 30, 54 and 78 months of child age. Assessments include questionnaires, observations, interviews and evaluations of home conditions, physical activity monitoring and the collection of buccal cells for genotyping. Regarding offspring development, we collect detailed information on temperament, social competence, neurodevelopment, and (early precursors of) psychopathology. In addition, we collect information on parenting and parental stress, parent-child interactions, self-efficacy, personality and parental psychopathology, family resources, and life events and difficulties. To date, over 300 children have been included in TRAILS-Next, and many more are expected to be included in the upcoming years

## 9 Conclusion

Intergenerational transmission takes place because of a complex interplay of genetic and non-genetic factors. To understand the underlying mechanisms of intergenerational transmission, longitudinal studies across multiple generations are needed with repeated measures of the traits and behaviors under examination and including information of both fathers and mothers. Genetically informative designs, including those with multiple offspring, the children-of-twins design, multiple genotyped generations or the virtual parent design, are needed to disentangle the contributions of genetic and nongenetic transmission and to understand interactions between genetic and nongenetic factors.

Together, the four large prospective longitudinal cohorts of Work Package 3 in the Consortium on Individual Development will advance earlier work by focusing on intergenerational transmission using longitudinal measures of parenting and psychopathology in each generation and designs based on information from transmitted and non-transmitted alleles. We will examine whether variation in parenting and psychopathology of Generation 3 depends on Generation 2 parents’ previous experiences as a child as reported by themselves as well as by their parents (Generation 1), and through which mechanisms this transmission occurs. Importantly, we will also examine the conditions under which parenting and psychopathology are *not* transmitted from one generation to another. In terms of mediating mechanisms of intergenerational transmission, we will particularly focus on social competence (Junge et al., current issue) and self-regulation (Vink et al., current issue), skills that are essential for functioning in society and for reducing the risk of psychopathology. Other strengths of the consortium include the use of genotype data for Mendelian randomization studies, to establish causality and the collection of epigenetic data.

As can be seen in Table 1, all studies try to assess confounding variables, including broader contextual data, rely on a longitudinal design, have prospective G2 and G3 data, and measured the concepts of interest in both G2 and G3 participants, assessed multiple children within the same family in at least one generation, and included both fathers and mothers. In addition, the four studies come with unique methodological strengths. Notable strengths are for instance the twin design of NTR and the prenatal measures in Generation R and TRAILS. RADAR and TRAILS have prospective data on parenting practices of two generations of parents and are therefore, amongst others, particularly suitable to examine intergenerational transmission of parenting Generation R also has data on two generation of parents, but G1 parents are only assessed during grandparenthood. A current limitation of the cohorts is that there are no assessments of G2 and G3 behavior in the same developmental stage, which has been identified as a key design criterium for studies on intergenerational transmission (Thornberry, 2016). However, this is a matter of time, as most of the G3 children are currently in early childhood.

Although each of the four cohorts uses slightly different approaches and measures, within the Consortium on Individual Development the methodology of Bayesian research synthesis has been developed, which allows to examine research questions using multiple cohorts and to aggregate the results over cohorts. This method has been applied successfully to examine the role of parental age in child psychopathology and neurodevelopmental outcomes (Zondervan-Zwijnenburg et al., 2019; Veldkamp et al., 2020) and provides a fruitful way to replicate and integrate results across cohorts. Using methods like this allows to test for the robustness of findings across studies, for example on gene-environment interactions, which tend to be quite inconsistent across individual studies. By combining the strengths of the individual cohort studies, it will be possible to provide a richer and more in-depth understanding of the processes of intergenerational transmission.

## Author contribution

SB drafted the manuscript. SG, ELdeZ, AMO, MEK-V, and DIB wrote sections of the manuscript. All other authors commented on the draft of this manuscript.

## Declaration of Competing Interest

The authors report no declarations of interest.
